# Changes in parental attitudes toward attention‐deficit/hyperactivity disorder impairment over time

**DOI:** 10.1002/jcv2.12238

**Published:** 2024-04-30

**Authors:** Miguel Garcia‐Argibay, Ralf Kuja‐Halkola, Sebastian Lundström, Paul Lichtenstein, Samuele Cortese, Henrik Larsson

**Affiliations:** ^1^ School of Medical Sciences Faculty of Medicine and Health Örebro University Örebro Sweden; ^2^ Department of Medical Epidemiology and Biostatistics Karolinska Institutet Stockholm Sweden; ^3^ Centre for Innovation in Mental Health School of Psychology Faculty of Environmental and Life Sciences University of Southampton Southampton UK; ^4^ Gillberg Neuropsychiatry Centre Institute of Neuroscience and Physiology University of Gothenburg Gothenburg Sweden; ^5^ Region Skåne, Psychiatry, Habilitation & Aid Child and Adolescent Psychiatry Regional Inpatient Care Emergency Unit Malmö Sweden; ^6^ Clinical and Experimental Sciences (CNS and Psychiatry) Faculty of Medicine University of Southampton Southampton UK; ^7^ Solent NHS Trust Southampton UK; ^8^ Hassenfeld Children's Hospital at NYU Langone New York University Child Study Center New York New York USA; ^9^ DiMePRe‐J‐Department of Precision and Regenerative Medicine‐Jonic Area University of Bari “Aldo Moro” Bari Italy

**Keywords:** ADD, ADHD, attention deficit hyperactivity disorder, epidemiology, prevalence

## Abstract

**Background:**

Over the last decades, the prevalence of Attention‐deficit/hyperactivity disorder (ADHD) has increased. However, the underlying explanation for this increase remains unclear. We aimed to assess whether there has been a secular change in how parents perceive the impairment conferred by ADHD symptomatology.

**Methods:**

Data for this study were obtained from the Child and Adolescent Twin Study in Sweden, involving 27,240 individuals whose parents answered a questionnaire when the children were 9 years old. We assessed the relationship between parentally perceived impairment caused by ADHD symptoms scores over time. The analysis was performed separately for five different birth cohorts, spanning three‐year periods from 1995 to 2009 and for ADHD inattention and hyperactivity/impulsivity dimensions.

**Results:**

We found a consistent upward trend of parents reporting impairment in relation to ADHD symptomatology across birth cohorts. Over a 12‐year period, comparing those born 2007–2009 (assessed 2016–2018) with those born 1995–1997 (assessed 2004–2006), impairment scores increased by 27% at clinically relevant levels of ADHD symptomatology. Notably, when specifically evaluating the hyperactivity/impulsivity dimension, the disparity was even more striking, with an increase of up to 77%.

**Conclusions:**

This study revealed a significant secular change in parental perception of impairment attributed to ADHD symptomatology over recent decades, providing new insights into the increased prevalence of ADHD. It underscores the need to better understand the factors that have contributed to the increased perception of impairment related to ADHD symptoms.

## Graphical abstract

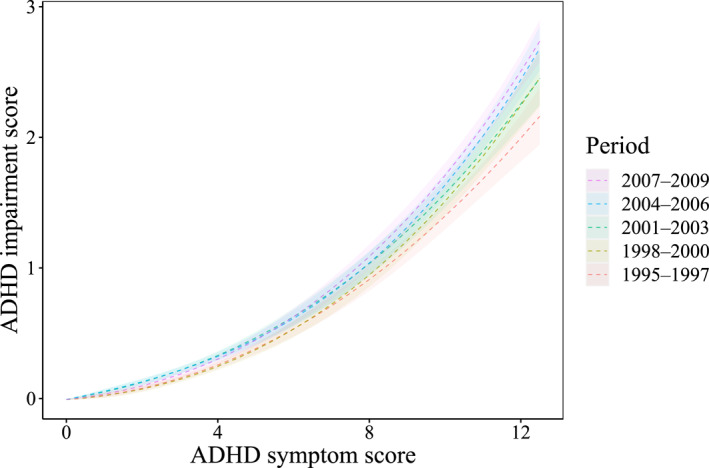

**Key points**

- Attention‐deficit/hyperactivity disorder (ADHD) diagnosis rates and treatment increased substantially over recent decades across multiple countries, while ADHD symptoms have remained stable in the population.
- Results showed a significant shift in parental perception of the impact of ADHD symptoms on their offspring, which may partially explain the increasing prevalence of ADHD over the past decades.
- These findings highlight the multidimensional factors influencing ADHD diagnosis rates.
- The evidence underscores the need for a balanced, holistic approach to ADHD assessment that combines careful consideration of parental perspectives with objective symptom measurement.

## INTRODUCTION

Attention Deficit‐Hyperactivity/Disorder (ADHD) is a neurodevelopmental disorder characterized by developmentally inappropriate levels of inattention and/or hyperactivity/impulsivity that frequently co‐occurs with other psychiatric and physical conditions (Faraone et al., 2021; Garcia‐Argibay et al., 2022a; Garcia‐Argibay et al., 2022b; Garcia‐Argibay, Li, et al., 2023; Garcia‐Argibay, Zhang‐James, et al., 2023; Li et al., 2022; Wendt et al., 2022). The estimated global prevalence of ADHD is approximately 5% in children and adolescents (Faraone et al., 2021). However, there is growing concern regarding the increasing rates of ADHD diagnoses and medication treatment over the past few decades (Cortese, Song, et al., 2023). Numerous studies worldwide consistently documented substantial increases over recent decades in the rates of clinically diagnosed and pharmacologically treated ADHD. For instance, research shows the 1‐year prevalence of diagnosed ADHD rose 3–7 fold across Nordic countries from 1990 to 2007 and over 25 fold in Taiwan from 1996 to 2005 (Atladottir et al., 2015). In the United States, the percentage of children diagnosed with ADHD increased from 6.1% in 1997 to 10.2% in 2015–2016 (Xu et al., 2018). Similarly, multiple studies demonstrated a marked rise in ADHD medication use in European countries and the US over a similar time period (Chan et al., 2023).

While clinically diagnosed rates and treatment for ADHD have increased over time, prior evidence indicated that ADHD symptoms have remained stable in the population (Rydell et al., 2018). This suggests that the surge in clinical ADHD prevalence may not reflect an actual increase in the underlying neurodevelopmental symptoms, but a confluence of medical and societal factors. Firstly, over the past three decades, the diagnostic criteria for ADHD have undergone successive changes due to the evolution of classification systems (Faraone et al., 2003) (e.g., the Diagnostic and Statistical Manual of Mental Disorders [DSM]). For example, the criteria were broadened with the release of the DSM‐IV in 1994 and further expanded with the DSM‐5 in 2013. These revisions to the clinical guidelines have resulted in a more inclusive definition of ADHD over time (Epstein & Loren, 2013) and are likely to have influenced diagnostic practices and contributed to an increase in ADHD diagnoses (Epstein & Loren, 2013). Second, increased awareness and recognition of ADHD may have contributed to the rise in diagnosis rates (Abdelnour et al., 2022; Castle et al., 2007). As more information about ADHD becomes available to the general public, parents and teachers may become more attuned to identifying symptoms in children and pursuing formal diagnosis. Third, changes in the social and educational environments may also have contributed to the rise in ADHD diagnosis rates. For example, the increasing demands of the modern educational system could spur overidentification of ADHD symptoms in struggling students as a response to the stress and pressure of school performance (Hinshaw, 2018). Fourth, another social factor potentially contributing to rising ADHD diagnoses might be the need, requested by many healthcare and educational systems, for a formal diagnosis for additional resource allocation and support. In fact, in order to receive academic accommodation, access certain medications, or qualify for therapy and other auxiliary services, a medical diagnosis is often required. This creates an incentive for parents and doctors to formally diagnose ADHD even in borderline cases where impairment may be modest. Additionally, awareness of ADHD as a medical condition rather than just "bad behavior" has grown substantially in recent decades. This reduces stigma and makes parents more likely to seek a diagnosis. In summary, the requirement of a diagnosis to receive interventions, combined with reduced stigma, may fuel diagnosis rates by motivating help‐seeking among parents. While this is of benefit to those children needing support, it may also lead to overidentification in mild cases.

Lastly, an important yet underexplored aspect potentially accounting for the observed rise in ADHD diagnosis rates revolves around the concept of significant symptoms‐related impairment as a diagnostic criterion for ADHD. Indeed, current diagnostic criteria for ADHD require evidence that symptoms interfere with, or reduce the quality, in school, work, or relationships (American Psychiatric Association, 2013). Therefore, shifts in parental perception of ADHD‐related impairment and dysfunction, stemming from the manifestation of ADHD symptoms, might play an important role in this phenomenon. A changing parental perspective concerning what constitutes dysfunction could potentially lead to a higher likelihood of ADHD diagnoses even if the symptom load has not increased. As societal attitudes, stigmatization, and awareness surrounding ADHD continue to evolve, parents might become more attuned to subtle manifestations of ADHD‐related difficulties in their children's lives. What might have been perceived as typical childhood behavior in the past, such as rambunctiousness, could now be regarded as constituting impairment and increasingly interpreted as symptoms warranting clinical attention. Consequently, a changing parental perspective regarding the impact of ADHD symptoms on various domains of functioning could potentially contribute to the increasing prevalence of ADHD.

In this study, we leveraged a comprehensive nationwide study covering a span of 12 consecutive years to examine, for the first time, potential changes in parental perception of ADHD impairment due to ADHD symptomatology over the past decades.

## METHODS

This study was approved by the Karolinska Institutet Ethical Review Board (Dnr 02–289 and 2018/960‐31‐2). The Strengthening the Reporting of Observational studies in Epidemiology (STROBE) guidelines were followed (Supporting Information S1: Appendix S1; von Elm et al., 2007).

### Study design and participants

We drew on data from The Child and Adolescent Twin Study in Sweden (CATSS). CATSS started in 2004, when parents of all twins in Sweden were invited to participate when the twin turned 9–12 years, with a response rate of 80% (see Anckarsäter et al., 2011 for a detailed description). We included all twins born between January 1, 1995, and December 31, 2009, all of whom were contacted when they turned 9 years old. After exclusions due to missing data on the ADHD rating scale, the cohort for the present study comprised 27,440 individuals, with only those having complete information being included.

### Measurements

We included questionnaire data from the Autism‐Tics, ADHD and other Comorbidities inventory (A‐TAC; Hansson et al., 2005), which is a comprehensive screening tool covering the most common child and adolescent psychiatric disorders. A‐TAC includes 96 questions of which 19 correspond to ADHD symptoms (9 items related to DSM‐5 inattention symptoms and 10 items to hyperactivity/impulsivity symptoms). Each question is scored 1 for “*yes*,” 0.5 for “*yes, to some extent*,” and 0 for “*no*,” giving a sum score ranging from 0 to 19 (henceforth called ADHD symptom score). Consistently with previous studies, we used two validated cutoffs for the ADHD symptom score: 6 (corresponding to a broad screening diagnosis) and 12.5 (used as a validated proxy for clinical diagnoses of ADHD; Mårland et al., 2017) The psychometric properties of the A‐TAC have been reported to be excellent, with an intra‐ and inter‐rater reliability of 0.89 and 0.84, respectively, and a Cronbach *α* of 0.92 (Larson et al., 2014). Furthermore, A‐TAC includes 17 items that address autism spectrum disorder (ASD)‐related domains (6 for language, 6 for social interaction, and 5 for flexibility), and 3 items are related to learning disabilities. The ASD module, with a cutoff score of ≥8.5, demonstrated a sensitivity of 0.30 and a specificity of 0.99. The learning disabilities subscale, validated against the ICD‐10 definitions of intellectual disability (F70‐F79), reported a sensitivity of 0.39 and a specificity of 0.99 (Larson et al., 2010; Mårland et al., 2017).

For each ADHD dimension (i.e., inattention and hyperactivity/impulsivity) where at least one item was scored 0.5 or 1, parents were questioned about the impact of endorsed symptoms on their child's functioning in various domains, including school, social interactions, and home (“*have the endorsed symptoms led to dysfunction at school, among peers, or at home?*”), and whether their child suffers from the symptoms themselves (“*does the child suffers from the symptoms?*”). The scoring procedure for these items follows the same methodology as the symptom scores. The cumulative score for each dimension ranged from 0 to 2, while the total cumulative score for the two dimensions ranged from 0 to 4. This comprehensive score is subsequently referred to as the ADHD impairment score. To investigate time‐trends, we grouped individuals into five birth cohorts: 1995–1997, 1998–2000, 2001–2003, 2004–2006, and 2007–2009.

### Statistical analysis

For descriptive purposes, ADHD symptom scores were grouped into five categories: 0, 0.5–4, 4.5–8, 8.5–12, and ≥12.5. ADHD impairment scores were then summarized within each endorsed ADHD symptoms category and further stratified by birth cohort.

We followed the methodology previously used when assessing the association between autism symptom score and related impairment (Lundström et al., 2022). To assess whether there was a secular change in how parents perceive the impairment conferred by ADHD symptomatology, we fitted a third‐degree polynomial regression model using an identity link, with ADHD symptoms scores as the independent variable and ADHD impairment score as the dependent variable. Further, we employed the same model to estimate the ratio of the modeled ADHD impairment score for each birth category relative to the 1995–1997 birth cohort. This allowed us to determine the relative increase in ADHD perceived impairment score per unit increase in ADHD symptom score. Cluster‐robust standard errors were used to account for within‐twin pair correlations and deviations from homoscedasticity. Analyses were stratified by ADHD dimension (i.e., inattention and hyperactivity/impulsivity) and sex. Due to the small number of individuals with higher ADHD symptom scores, scores were truncated to the cut‐off of the validated proxy for clinical diagnoses of ADHD of 12.5 (Mårland et al., 2017). All analyses were performed using R version 4.2.3 (R Core Team, 2020). Analyses were conducted between May 5, 2023, and June 22, 2023.

### Sensitivity analysis

We assessed the appropriateness of a cubic approximation of the ADHD symptom score for predicting the ADHD impairment score by plotting the regression lines of the cubic model against (a) the observed mean impairment score with its confidence interval, and (b) a local polynomial regression line (with arguably better local fit to the data). Analyses were performed separately for each birth cohort to ensure adequate fit to the data across different birth cohorts. By comparing the fitted regression lines of the cubic model with the observed mean impairment score, along with its confidence interval, and a local polynomial regression line, we were able to determine if the model adequately captures the relationship between the symptom score and impairment. Lastly, to ascertain the robustness of our conclusions and assess the potential impact of a different threshold, we conducted a sensitivity analysis by increasing the truncation cut‐off value to 14.5.

### Role of the funding source

The funders had no role in the design and conduct of the study; collection, management, analysis, and interpretation of the data; preparation, review, or approval of the manuscript; and decision to submit the manuscript for publication.

## RESULTS

The cohort for this study comprised 27,440 twins, of which 13,898 (51%) were boys. Among those, 3272 (11.9%) and 685 (2.5%) individuals had ADHD symptom scores exceeding high and low diagnostic thresholds respectively, 435 (1.6%) exceeded thresholds for learning disabilities, and 352 (1.3%) had symptom levels indicative of ASD. Overall, compared with females, a higher proportion of boys had ADHD symptom scores of 0.5 or higher and the disparity between sexes increased as ADHD symptom scores increased. Mean ADHD impairment scores showed an upward trend for symptom scores of 4.5 or higher in the later birth cohorts (Table 1).

**TABLE 1:** Descriptive information of the cohort by ADHD symptom score (0, 0.5–4, 4.5–8, 8.5–12, and ≥12.5).

Characteristic	Overall (*N* = 27,440)	ADHD symptom score				
		0 (*N* = 10,277)	0.5–4.0 (*N* = 12,287)	4.5–8.0 (*N* = 3112)	8.5–12.0 (*N* = 1079)	≥12.5 (*N* = 685)
Sex, *n* (%)						
Males	13,898 (51)	4443 (43)	6337 (52)	1920 (62)	711 (66)	487 (71)
Females	13,542 (49)	5834 (57)	5950 (48)	1192 (38)	368 (34)	198 (29)
ADHD impairment score, mean (SD)	0.23 (0.69)	0.00 (0.00)	0.08 (0.34)	0.61 (0.86)	1.51 (1.17)	2.60 (1.22)
Birth cohort, *n* (%)						
1995–1997	6047 (22)	2580 (25)	2540 (21)	641 (21)	167 (15)	119 (17)
1998–2000	5673 (21)	2425 (24)	2357 (19)	591 (19)	192 (18)	108 (16)
2001–2003	5776 (21)	2201 (21)	2651 (22)	607 (20)	190 (18)	127 (19)
2004–2006	4997 (18)	1592 (15)	2412 (20)	603 (19)	253 (23)	137 (20)
2007–2009	4947 (18)	1479 (14)	2327 (19)	670 (22)	277 (26)	194 (28)
ADHD impairment score by birth cohort, mean (SD)						
1995–1997	0.17 (0.57)	0 (0)	0.07 (0.32)	0.56 (0.81)	1.26 (1.10)	2.24 (1.30)
1998–2000	0.18 (0.61)	0 (0)	0.06 (0.30)	0.54 (0.80)	1.52 (1.23)	2.45 (1.28)
2001–2003	0.22 (0.65)	0 (0)	0.10 (0.36)	0.66 (0.87)	1.42 (1.15)	2.54 (1.24)
2004–2006	0.28 (0.75)	0 (0)	0.10 (0.37)	0.67 (0.87)	1.56 (1.10)	2.81 (1.10)
2007–2009	0.33 (0.85)	0 (0)	0.09 (0.36)	0.64 (0.92)	1.67 (1.21)	2.81 (1.16)

**FIGURE 1:**
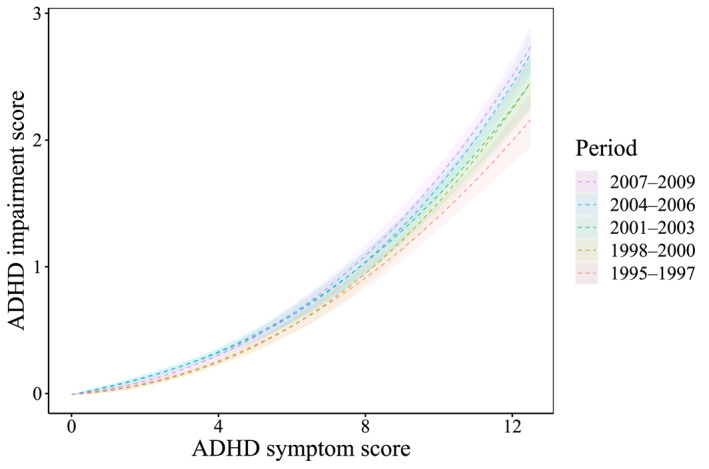
Association between ADHD symptom score and ADHD impairment score.

**FIGURE 2:**
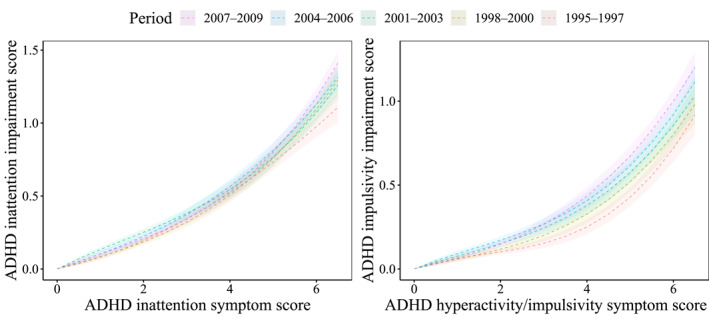
Association between ADHD inattention and hyperactivity/impulsivity symptom score with ADHD impairment score.

When assessing the relationship between ADHD symptoms and impairment, we observed an increased modeled ADHD impairment score for all birth cohorts (Figure 1). Summaries of the fitted regression coefficients and modeled ADHD impairment scores are presented in Tables S1 and S2, respectively. Table 2 displays the pairwise comparisons between the different birth cohorts. Notably, these differences became more evident when examining ADHD dimensions individually, revealing that modeled hyperactivity/impulsivity scores exhibited the largest increase across all birth cohorts (Figure 2).

**TABLE 2:** Pairwise comparisons between different birth cohorts.

	1998–2000	2001–2003	2004–2006	2007–2010	*p*_overall_
Overall					
1995–1997	0.25	<0.001	<0.001	<0.001	
1998–2000		<0.001	0.04	0.30	
2001–2003			0.49	0.04	
2004–2006				0.30	<0.001
Inattention					
1995–1997	0.08	<0.001	0.003	<0.001	
1998–2000		0.10	0.005	0.28	
2001–2003			0.14	0.005	
2004–2006				0.28	<0.001
Hyperactivity/impulsivity					
1995–1997	0.12	<0.001	<0.001	<0.001	
1998–2000		<0.001	0.05	0.09	
2001–2003			0.28	0.05	
2004–2006				0.09	<0.001

*Note*: *p*_overall_ is the *p*‐value for all estimates being the same.

As the birth cohorts progressed from 1995–1997 to 2007–2009, there was a gradual increase in the ADHD parent‐perceived impairment across most ADHD symptom score levels, with the largest differences for hyperactivity/impulsivity (Tables S3–S5). Specifically, compared with the earliest birth cohort, the latest cohort displayed on average a 17% higher impairment score at an ADHD symptom score of 6, and a 27% higher ratio at a symptom score of 12.5. When examining each ADHD subscale separately, we observed an increased ratio for low and high ADHD symptom scores in the inattention dimension, with similar values between boys and girls. This increase was also larger in the hyperactivity/impulsivity dimension (Tables S6–S11), with a 73% higher impairment score at an ADHD symptom score of 3, and a 31% higher ratio at a symptom score of 6. The results of the sensitivity analysis indicated that the cubic function provided a good fit to the data (Figure S1) and that ADHD impairment score ratio yielded comparable insights when using a truncation cut‐off of 14.5 (Table S12).

## DISCUSSION

This study aimed to explore for the first time whether there has been a secular change in parental perception of the functional impairments attributed to ADHD symptomatology in their offspring. Our findings provided evidence of a temporal shift, revealing a significant increase in the proportion of parents reporting impairment related to ADHD symptomatology. This finding may contribute to explaining the increasing prevalence of ADHD.

The underlying mechanisms driving the shift in parental perception of ADHD symptomatology remains unclear, but we propose several complementary explanations. First, it is plausible that ADHD symptoms may indeed be more impairing in the current societal context. Factors such as the pervasive use of digital devices (Shuai et al., 2021), increased academic pressure (Owens, 2021), and changes in parenting styles (Bhide et al., 2019) may exacerbate the challenges faced by children with ADHD symptoms, resulting in more pronounced levels of perceived impairment compared to a decade ago. For example, a shift toward more permissive, lenient parenting that places fewer demands on children could lead to less structure and routine in children's lives, potentially exacerbating ADHD symptoms like impulsivity and difficulty with organization. Increased digital device use can lead to shortened attention spans (Santos et al., 2022), exacerbating existing ADHD tendencies (Tamana et al., 2019).

Second, the upward trend in the proportion of parents reporting impairment may in part be due to the increased awareness of ADHD. This rise in awareness and reporting of ADHD can be attributed to multiple factors, such as increased media attention, changes in diagnostic criteria, and improved accessibility to diagnostic and treatment services. Greater public awareness of ADHD may shape parental expectations regarding child behavior. Changing perceptions in parents about what constitutes impairment from ADHD behaviors in children could influence diagnosis trends. If parents now believe that behaviors once considered typical childhood conduct are unacceptable and impairing, this evolving attitude could translate into higher rates of children referred for ADHD evaluation and subsequent diagnosis. Therefore, parents today may have lower thresholds for perceiving dysfunction and adversity in their children from typical ADHD symptoms like inattention or hyperactivity compared to past generations.

Our finding of a secular change in parental perception of the functional impairments and subjective distress attributed to ADHD symptomatology may suggest that the measurement of functional impairment in ADHD needs further considerations. Traditionally, impairment has been assessed using subjective reports from parents, teachers, and clinicians. However, there is increasing recognition of the importance of using objective measures and incorporating multiple perspectives to capture the multifaceted nature of impairment (Bölte et al., 2018). Objective measures, such as performance‐based assessments, direct observation, and ecological momentary assessment, could offer valuable insights into the real‐world impact of ADHD symptoms on various domains of functioning. However, objective measures currently do not have adequate sensitivity or specificity for neurodevelopmental disorders, highlighting the need for clinical experience and clinicians well‐versed in child development (Cortese, Solmi, et al., 2023). Integrating both clinical expertise and objective measures provides a more comprehensive and accurate assessment of functional impairment in ADHD. Clinicians should take into account the broader context of a child's life, including their functioning at school, within peer relationships, and at home. By adopting a holistic approach, clinicians can obtain a more comprehensive understanding of the child's overall impairment, beyond the perspective provided by parents, and tailor treatment plans accordingly. Furthermore, the study emphasizes clinicians should be aware that parent‐perceived impairment is not rated consistently across birth cohorts. Future research should continue to investigate the impact of ADHD on families and explore potential factors that may contribute to changes in parental perception over time.

Notably, similar patterns of increasing parent‐perceived impairment over time have emerged across other conditions beyond ADHD. For instance, a recent Swedish study (Lundström et al., 2022) found increased parent‐reported impairment associated with autism symptoms across multiple birth cohorts. Given these parallels, it is of the essence to overhaul the social milieu that children today experience in order to reveal the possible societal mechanisms that drive the shift in parental perception of impairment. Such studies hold the potential to develop interventions and environments aimed at alleviating impairment, thus reducing the need for psychiatric assessments. While ADHD and ASD serve as salient case studies for shifting parental perceptions of child psychopathology, future studies should examine whether related patterns hold for other common childhood mental conditions like anxiety, conduct disorders, obsessive compulsive disorders or specific learning disabilities. It is important to determine if surveillance biases are isolated to attention and activity levels or whether reduced tolerance levels, projecting adult stress mindsets, and appellation of childhood struggles as “disorders” are emblematic of more sweeping attitudinal transformations. Broader diagnostic trend analyses tied to changing social norms would prove informative.

This study had some limitations that should be considered when interpreting the results. First, as our sample comprised only Swedish twins, the generalizability of our findings to other populations is limited. Understanding the observed trends within a cross‐cultural lens is crucial, as cultural attitudes and expectations can significantly influence the perception and diagnosis of ADHD. For instance, symptom thresholding, stigma, and healthcare access issues likely yield underdiagnosis in other regions (Chan et al., 2022). Comparative, longitudinal analyses accounting for cultural attitudes and diagnostic norms could elucidate actual international variability versus artifacts of evolving societal factors and medical systems. Second, our study relied solely on subjective parental reports of ADHD symptomatology and associated impairment, which may be subject to bias and error. Furthermore, it is important to investigate whether similar trends in rating thresholds are observed among teachers and clinicians across different countries and cultures. Studies exploring changes in their perceptions of ADHD symptoms over time could provide valuable insights into potential cultural influences and broader societal shifts in how child behavior is viewed. Third, the study was cross‐sectional in nature and did not examine changes in perception over time within individuals. Finally, it should be noted that our study did not examine objective measures of impairment, and therefore further research is needed to determine if these results hold up when using more objective measures.

In summary, this study reveals evolving parental perceptions of ADHD‐related impairment that underscore the need for clinicians to adapt their diagnostic and treatment approaches accordingly. While incorporating parent insights provides crucial information, clinicians should balance this with more objective assessment of actual symptom severity and impairment. Over‐relying on shifting subjective attitudes risks misaligning clinical care with children's true needs. By combining careful parental reporting with additional measures (e.g., cognitive testing, teacher input), clinicians can gain a more accurate understanding of patients' strengths, challenges, and support requirements.

## AUTHOR CONTRIBUTIONS

Conceptualization: Miguel Garcia‐Argibay, Sebastian Lundström, Henrik Larsson. Formal analysis: Miguel Garcia‐Argibay. Funding acquisition: Henrik Larsson. Investigation: Miguel Garcia‐Argibay. Methodology: Miguel Garcia‐Argibay, Ralf Kuja‐Halkola. Project administration: Miguel Garcia‐Argibay, Henrik Larsson. Resources: Henrik Larsson. Supervision: Henrik Larsson. Visualization: Miguel Garcia‐Argibay. Writing, original draft: Miguel Garcia‐Argibay. Writing, review & editing: All authors.

## CONFLICT OF INTEREST STATEMENT

Henrik Larsson reported receiving grants from Shire/Takeda Pharmaceuticals during the conduct of the study; personal fees from and serving as a speaker for Shire/Takeda Pharmaceuticals and Evolan Pharma AB outside the submitted work; and sponsorship for a conference on ADHD from Shire Pharmaceuticals outside the submitted work. The remaining authors declare having no conflict of interest.

## ETHICS STATEMENT

This study was approved by the Karolinska Institute Ethical Review Board (Dnr 02–289 and 2018/960‐31‐2).

## Supporting information

Supporting Information S1

## Data Availability

The Public Access to Information and Secrecy Act in Sweden prohibits us from making individual level data publicly available. Researchers who are interested in replicating our work can apply for individual level data at Statistics Sweden: www.scb.se/en/services/guidance‐for‐researchers‐and‐universities/.
