# Supplementary material for: Changes in parental attitudes toward attention‐deficit/hyperactivity disorder impairment over time
Source: JCPP Adv. 2024 Apr 30;4(3):e12238. doi: 10.1002/jcv2.12238 (PMC11472822; doi:10.1002/jcv2.12238)
Supplement: Supplementary file 1 — Supporting Information S1 [file JCV2-4-e12238-s001.docx]

**Appendix for:**

**Changes in parental attitudes towards attention-deficit/hyperactivity disorder impairment over time**

Miguel Garcia-Argibay^1,2*^, Ph.D., Ralf Kuja-Halkola^2^, Ph.D., Sebastian Lundström^3^, Ph.D., Paul Lichtenstein^2^, Ph.D., Samuele Cortese^4,5,6,7,8^, MD, Ph.D., Henrik Larsson^1,2^, Ph.D.

# Contents

[Contents 1](#_Toc147746883)

[Table S1. Summary of the regression coefficients with cluster-robust standard errors (SE). 2](#_Toc147746884)

[Table S2. Summary of the predicted ADHD impairment scores with 95% confidence intervals by birth cohort. 3](#_Toc147746885)

[Table S3. Summary for the ratios of the modeled ADHD impairment scores with 95% confidence intervals. 4](#_Toc147746886)

[Table S4. Summary for the ratios of the modeled ADHD inattention impairment scores with 95% confidence intervals. 5](#_Toc147746887)

[Table S5. Summary for the ratios of the modeled ADHD hyperactivity/impulsivity impairment scores with 95% confidence intervals. 6](#_Toc147746888)

[Table S6. Summary for the ratios of the modeled ADHD impairment scores for males with 95% confidence intervals. 7](#_Toc147746889)

[Table S7. Summary for the ratios of the modeled ADHD inattention impairment scores for males with 95% confidence intervals. 8](#_Toc147746890)

[Table S8. Summary for the ratios of the modeled ADHD hyperactivity/impulsivity impairment for males scores with 95% confidence intervals. 8](#_Toc147746891)

[Table S9. Summary for the ratios of the modeled ADHD impairment scores for females with 95% confidence intervals. 9](#_Toc147746892)

[Table S10. Summary for the ratios of the modeled ADHD inattention impairment scores for females with 95% confidence intervals. 10](#_Toc147746893)

[Table S11. Summary for the ratios of the modeled ADHD hyperactivity/impulsivity impairment for females scores with 95% confidence intervals. 10](#_Toc147746894)

[Table S12. Summary for the ratios of the modeled ADHD scores with 95% confidence intervals with ADHD symptoms scores truncated at 14.5. 11](#_Toc147746895)

[Figure S1. Comparisons of model fit between a cubic model and a local polynomial. For each birth cohort there is a mean ADHD impairment score with 95% confidence interval per ADHD symptom level. 12](#_Toc147746896)

# Table S1. Summary of the regression coefficients with cluster-robust standard errors (SE).

| **Parameter** | ***b*** | ***SE*** | ***z*** | ***p*** |
| --- | --- | --- | --- | --- |
| Intercept (μ) | -0·01 | 0·002 | -5·12 | <·0001 |
| ADHD symptom score × Birth year 1995–1997 | 0·02 | 0·013 | 1·58 | ·113 |
| ADHD symptom score × Birth year 1998–2000 | 0·02 | 0·014 | 1·48 | ·139 |
| ADHD symptom score × Birth year 2001–2003 | 0·05 | 0·014 | 3·72 | ·0002 |
| ADHD symptom score × Birth year 2004–2006 | 0·06 | 0·014 | 4·41 | <·0001 |
| ADHD symptom score × Birth year 2007–2009 | 0·03 | 0·014 | 2·22 | ·026 |
| Birth year 1995–1997 × ADHD symptom score^2^ | 0·01 | 0·005 | 2·38 | ·017 |
| Birth year 1998–2000 × ADHD symptom score^2^ | 0·01 | 0·005 | 1·91 | ·056 |
| Birth year 2001–2003 × ADHD symptom score^2^ | 0·01 | 0·005 | 1·39 | ·164 |
| Birth year 2004–2006 × ADHD symptom score^2^ | 0·002 | 0·005 | 0·54 | ·589 |
| Birth year 2007–2009 × ADHD symptom score^2^ | 0·01 | 0·005 | 2·11 | ·035 |
| Birth year 1995–1997 × ADHD symptom score^3^ | 0·0001 | 0·0003 | 0·30 | ·764 |
| Birth year 1998–2000 × ADHD symptom score^3^ | 0·0004 | 0·0003 | 1·15 | ·249 |
| Birth year 2001–2003 × ADHD symptom score^3^ | 0·0004 | 0·0003 | 1·20 | ·228 |
| Birth year 2004–2006 × ADHD symptom score^3^ | 0·0008 | 0·0003 | 2·58 | ·009 |
| Birth year 2007–2009 × ADHD symptom score^3^ | 0·0004 | 0·0003 | 1·28 | ·199 |

Note: ‘×’ indicates a modeled statistical interaction. The regression model is

$$E\left( y | x,\boldsymbol{z} \right)=\mu+\beta_{1}x\cdot z_{1}+\beta_{2}x\cdot z_{2}+\beta_{3}x\cdot z_{3}+\beta_{4}x\cdot z_{4}+\beta_{5}x\cdot z_{5}+\beta_{6}x^{2}\cdot z_{1}+\beta_{7}x^{2}\cdot z_{2}+\beta_{8}x^{2}\cdot z_{3}+\beta_{9}x^{2}\cdot z_{4}+\beta_{10}x^{2}\cdot z_{5}+ \beta_{11}x^{3}\cdot z_{1}+\beta_{12}x^{3}\cdot z_{2}+\beta_{13}x^{3}\cdot z_{3}+\beta_{14}x^{3}\cdot z_{4}+\beta_{15}x^{3}\cdot z_{5}$$

Where $y$ is ADHD impairment score, $x$ is ADHD symptom score, $z_{1},z_{2},z_{3},z_{4}$, and $z_{5}$ are binary indicators of cohort 1995–1997, 1998–2000, 2001–2003, 2004–2006, and 2007–2009.

# Table S2. Summary of the predicted ADHD impairment scores with 95% confidence intervals by birth cohort.

|  | **Birth cohort** | | | | |
| --- | --- | --- | --- | --- | --- |
| **ADHD symptom score** | **1995–1997** | **1998–2000** | **2001–2003** | **2004–2006** | **2007–2009** |
| 0 | -0·01 (-0·01-0·00) | -0·01 (-0·01-0·00) | -0·01 (-0·01-0·00) | -0·01 (-0·01-0·00) | -0·01 (-0·01-0·00) |
| 0·5 | 0·01 (0·00-0·01) | 0·00 (-0·01-0·01) | 0·02 (0·01-0·03) | 0·02 (0·01-0·03) | 0·01 (0·00-0·02) |
| 1 | 0·02 (0·01-0·04) | 0·02 (0·00-0·04) | 0·05 (0·03-0·07) | 0·06 (0·04-0·07) | 0·03 (0·02-0·05) |
| 1·5 | 0·05 (0·03-0·07) | 0·04 (0·02-0·07) | 0·09 (0·06-0·11) | 0·09 (0·07-0·11) | 0·06 (0·04-0·08) |
| 2 | 0·08 (0·06-0·10) | 0·07 (0·05-0·09) | 0·12 (0·10-0·15) | 0·13 (0·10-0·15) | 0·10 (0·07-0·12) |
| 2·5 | 0·11 (0·09-0·14) | 0·11 (0·08-0·13) | 0·17 (0·14-0·19) | 0·17 (0·14-0·20) | 0·14 (0·11-0·16) |
| 3 | 0·16 (0·13-0·18) | 0·15 (0·12-0·17) | 0·22 (0·19-0·24) | 0·22 (0·19-0·24) | 0·19 (0·16-0·21) |
| 3·5 | 0·20 (0·18-0·23) | 0·19 (0·17-0·22) | 0·27 (0·24-0·30) | 0·27 (0·24-0·30) | 0·24 (0·21-0·27) |
| 4 | 0·26 (0·23-0·29) | 0·25 (0·22-0·27) | 0·33 (0·30-0·36) | 0·32 (0·29-0·36) | 0·30 (0·27-0·33) |
| 4·5 | 0·32 (0·28-0·35) | 0·31 (0·27-0·34) | 0·39 (0·36-0·43) | 0·39 (0·35-0·42) | 0·37 (0·33-0·41) |
| 5 | 0·38 (0·34-0·43) | 0·37 (0·33-0·42) | 0·47 (0·42-0·51) | 0·45 (0·41-0·50) | 0·45 (0·40-0·49) |
| 5·5 | 0·45 (0·40-0·51) | 0·45 (0·40-0·50) | 0·54 (0·49-0·60) | 0·53 (0·48-0·58) | 0·53 (0·48-0·59) |
| 6 | 0·53 (0·47-0·59) | 0·53 (0·47-0·59) | 0·63 (0·56-0·69) | 0·61 (0·55-0·67) | 0·63 (0·56-0·69) |
| 6·5 | 0·62 (0·55-0·69) | 0·62 (0·55-0·69) | 0·72 (0·64-0·79) | 0·71 (0·64-0·77) | 0·73 (0·66-0·80) |
| 7 | 0·71 (0·63-0·78) | 0·72 (0·64-0·80) | 0·81 (0·73-0·90) | 0·81 (0·73-0·88) | 0·84 (0·76-0·92) |
| 7·5 | 0·81 (0·73-0·89) | 0·83 (0·74-0·92) | 0·92 (0·83-1·01) | 0·92 (0·83-1·00) | 0·96 (0·87-1·04) |
| 8 | 0·91 (0·83-1·00) | 0·95 (0·86-1·04) | 1·03 (0·94-1·13) | 1·04 (0·95-1·12) | 1·09 (1·00-1·18) |
| 8·5 | 1·02 (0·93-1·11) | 1·07 (0·98-1·17) | 1·15 (1·06-1·25) | 1·17 (1·08-1·26) | 1·23 (1·13-1·32) |
| 9 | 1·14 (1·05-1·23) | 1·21 (1·11-1·31) | 1·28 (1·18-1·39) | 1·31 (1·22-1·40) | 1·38 (1·28-1·47) |
| 9·5 | 1·27 (1·17-1·36) | 1·36 (1·25-1·46) | 1·42 (1·32-1·53) | 1·47 (1·38-1·56) | 1·54 (1·44-1·63) |
| 10 | 1·40 (1·29-1·50) | 1·51 (1·40-1·62) | 1·57 (1·46-1·68) | 1·63 (1·54-1·73) | 1·71 (1·61-1·80) |
| 10·5 | 1·54 (1·42-1·65) | 1·68 (1·56-1·79) | 1·73 (1·61-1·84) | 1·82 (1·72-1·91) | 1·89 (1·79-1·99) |
| 11 | 1·68 (1·56-1·81) | 1·86 (1·73-1·98) | 1·89 (1·77-2·02) | 2·01 (1·91-2·11) | 2·08 (1·98-2·19) |
| 11·5 | 1·83 (1·69-1·98) | 2·04 (1·89-2·19) | 2·07 (1·93-2·21) | 2·22 (2·10-2·33) | 2·29 (2·18-2·40) |
| 12 | 1·99 (1·82-2·17) | 2·24 (2·06-2·42) | 2·26 (2·08-2·43) | 2·44 (2·30-2·58) | 2·51 (2·37-2·64) |
| 12·5 | 2·16 (1·94-2·38) | 2·45 (2·24-2·67) | 2·45 (2·25-2·66) | 2·68 (2·51-2·85) | 2·74 (2·57-2·90) |

# Table S3. Summary for the ratios of the modeled ADHD impairment scores with 95% confidence intervals.

|  | **Birth cohort** | | | | |
| --- | --- | --- | --- | --- | --- |
| **ADHD symptom score** | **1994–1997** | **1998–2000** | **2001–2003** | **2004–2006** | **2007–2009** |
| 0 | Reference | NA | NA | NA | NA |
| 0·5 | Reference | 0·88 (-0·81-2·56) | 3·84 (2·14-5·54) | 4·51 (2·80-6·23) | 1·98 (0·23-3·73) |
| 1 | Reference | 0·92 (0·31-1·53) | 2·13 (1·50-2·75) | 2·34 (1·70-2·97) | 1·41 (0·76-2·05) |
| 1·5 | Reference | 0·93 (0·56-1·29) | 1·78 (1·39-2·16) | 1·88 (1·49-2·28) | 1·30 (0·90-1·69) |
| 2 | Reference | 0·93 (0·69-1·17) | 1·60 (1·34-1·86) | 1·65 (1·38-1·92) | 1·24 (0·98-1·51) |
| 2·5 | Reference | 0·94 (0·77-1·10) | 1·48 (1·30-1·66) | 1·49 (1·30-1·69) | 1·21 (1·03-1·40) |
| 3 | Reference | 0·94 (0·82-1·06) | 1·39 (1·26-1·53) | 1·39 (1·24-1·54) | 1·19 (1·05-1·34) |
| 3·5 | Reference | 0·95 (0·85-1·05) | 1·33 (1·22-1·45) | 1·31 (1·19-1·44) | 1·18 (1·07-1·3) |
| 4 | Reference | 0·96 (0·86-1·05) | 1·28 (1·17-1·39) | 1·26 (1·15-1·37) | 1·18 (1·07-1·28) |
| 4·5 | Reference | 0·97 (0·87-1·06) | 1·25 (1·14-1·35) | 1·22 (1·11-1·32) | 1·17 (1·07-1·28) |
| 5 | Reference | 0·98 (0·87-1·08) | 1·22 (1·11-1·33) | 1·19 (1·08-1·29) | 1·17 (1·06-1·28) |
| 5·5 | Reference | 0·99 (0·88-1·09) | 1·19 (1·08-1·31) | 1·16 (1·05-1·28) | 1·17 (1·06-1·28) |
| 6 | Reference | 1·00 (0·89-1·11) | 1·17 (1·06-1·29) | 1·15 (1·04-1·26) | 1·17 (1·06-1·29) |
| 6·5 | Reference | 1·01 (0·89-1·12) | 1·16 (1·04-1·28) | 1·14 (1·03-1·26) | 1·18 (1·06-1·29) |
| 7 | Reference | 1·02 (0·90-1·13) | 1·15 (1·03-1·27) | 1·14 (1·02-1·25) | 1·18 (1·06-1·30) |
| 7·5 | Reference | 1·03 (0·91-1·14) | 1·14 (1·02-1·26) | 1·14 (1·02-1·25) | 1·19 (1·07-1·31) |
| 8 | Reference | 1·04 (0·93-1·15) | 1·13 (1·02-1·25) | 1·14 (1·02-1·25) | 1·19 (1·07-1·31) |
| 8·5 | Reference | 1·05 (0·94-1·16) | 1·13 (1·01-1·25) | 1·14 (1·03-1·26) | 1·20 (1·08-1·32) |
| 9 | Reference | 1·06 (0·95-1·17) | 1·13 (1·01-1·24) | 1·15 (1·04-1·26) | 1·21 (1·09-1·33) |
| 9·5 | Reference | 1·07 (0·96-1·18) | 1·12 (1·01-1·24) | 1·16 (1·05-1·27) | 1·21 (1·10-1·33) |
| 10 | Reference | 1·08 (0·97-1·19) | 1·12 (1·01-1·24) | 1·17 (1·06-1·28) | 1·22 (1·10-1·34) |
| 10·5 | Reference | 1·09 (0·97-1·21) | 1·12 (1·00-1·25) | 1·18 (1·06-1·30) | 1·23 (1·10-1·36) |
| 11 | Reference | 1·10 (0·97-1·24) | 1·13 (0·99-1·26) | 1·20 (1·06-1·33) | 1·24 (1·10-1·38) |
| 11·5 | Reference | 1·11 (0·96-1·27) | 1·13 (0·97-1·28) | 1·21 (1·05-1·37) | 1·25 (1·08-1·41) |
| 12 | Reference | 1·13 (0·94-1·31) | 1·13 (0·95-1·32) | 1·22 (1·03-1·42) | 1·26 (1·06-1·45) |
| 12·5 | Reference | 1·14 (0·91-1·36) | 1·13 (0·91-1·36) | 1·24 (1·00-1·48) | 1·27 (1·03-1·51) |

# Table S4. Summary for the ratios of the modeled ADHD inattention impairment scores with 95% confidence intervals.

|  | **Birth cohorts** | | | | |
| --- | --- | --- | --- | --- | --- |
| **ADHD symptom score** | **1994–1997** | **1998–2000** | **2001–2003** | **2004–2006** | **2007–2009** |
| 0 | Reference | NA | NA | NA | NA |
| 0·5 | Reference | 1·11 (0·82-1·41) | 1·91 (1·59-2·22) | 1·38 (1·08-1·68) | 1·44 (1·13-1·75) |
| 1 | Reference | 1·03 (0·84-1·22) | 1·64 (1·43-1·84) | 1·27 (1·07-1·47) | 1·26 (1·06-1·46) |
| 1·5 | Reference | 0·98 (0·86-1·10) | 1·44 (1·30-1·57) | 1·20 (1·06-1·33) | 1·14 (1·01-1·27) |
| 2 | Reference | 0·95 (0·86-1·04) | 1·29 (1·19-1·39) | 1·14 (1·04-1·24) | 1·06 (0·97-1·16) |
| 2·5 | Reference | 0·94 (0·86-1·02) | 1·18 (1·09-1·27) | 1·11 (1·02-1·20) | 1·02 (0·93-1·10) |
| 3 | Reference | 0·94 (0·85-1·02) | 1·11 (1·02-1·21) | 1·09 (1·00-1·18) | 1·00 (0·91-1·09) |
| 3·5 | Reference | 0·95 (0·86-1·04) | 1·07 (0·97-1·17) | 1·08 (0·99-1·18) | 1·00 (0·91-1·09) |
| 4 | Reference | 0·97 (0·88-1·06) | 1·04 (0·95-1·14) | 1·08 (0·99-1·18) | 1·02 (0·93-1·11) |
| 4·5 | Reference | 1·00 (0·92-1·09) | 1·04 (0·95-1·13) | 1·09 (1·00-1·18) | 1·05 (0·96-1·14) |
| 5 | Reference | 1·04 (0·96-1·12) | 1·05 (0·96-1·13) | 1·11 (1·03-1·19) | 1·09 (1·01-1·17) |
| 5·5 | Reference | 1·08 (1·00-1·15) | 1·07 (0·99-1·15) | 1·13 (1·05-1·21) | 1·14 (1·07-1·22) |
| 6 | Reference | 1·12 (1·03-1·21) | 1·10 (1·01-1·19) | 1·16 (1·07-1·24) | 1·21 (1·12-1·29) |
| 6·5 | Reference | 1·17 (1·04-1·30) | 1·14 (1·02-1·26) | 1·19 (1·07-1·31) | 1·27 (1·15-1·40) |

# Table S5. Summary for the ratios of the modeled ADHD hyperactivity/impulsivity impairment scores with 95% confidence intervals.

|  | **Birth cohorts** | | | | |
| --- | --- | --- | --- | --- | --- |
| **ADHD symptom score** | **1994–1997** | **1998–2000** | **2001–2003** | **2004–2006** | **2007–2009** |
| 0 | Reference | NA | NA | NA | NA |
| 0·5 | Reference | 0·86 (0·59-1·12) | 1·11 (0·85-1·37) | 1·36 (1·06-1·65) | 0·88 (0·60-1·17) |
| 1 | Reference | 0·95 (0·74-1·16) | 1·23 (1·02-1·44) | 1·47 (1·23-1·72) | 1·06 (0·83-1·29) |
| 1·5 | Reference | 1·05 (0·89-1·22) | 1·36 (1·20-1·53) | 1·59 (1·40-1·79) | 1·26 (1·07-1·44) |
| 2 | Reference | 1·16 (1·01-1·31) | 1·48 (1·33-1·63) | 1·69 (1·52-1·86) | 1·46 (1·29-1·62) |
| 2·5 | Reference | 1·25 (1·08-1·42) | 1·56 (1·39-1·74) | 1·75 (1·57-1·93) | 1·63 (1·45-1·80) |
| 3 | Reference | 1·31 (1·11-1·50) | 1·59 (1·39-1·79) | 1·75 (1·55-1·95) | 1·73 (1·54-1·93) |
| 3·5 | Reference | 1·32 (1·13-1·52) | 1·57 (1·37-1·77) | 1·70 (1·51-1·90) | 1·77 (1·57-1·97) |
| 4 | Reference | 1·30 (1·12-1·49) | 1·51 (1·32-1·70) | 1·63 (1·44-1·81) | 1·74 (1·55-1·92) |
| 4·5 | Reference | 1·27 (1·11-1·42) | 1·43 (1·27-1·59) | 1·53 (1·37-1·69) | 1·67 (1·51-1·83) |
| 5 | Reference | 1·22 (1·09-1·34) | 1·34 (1·21-1·48) | 1·44 (1·31-1·57) | 1·58 (1·44-1·71) |
| 5·5 | Reference | 1·16 (1·05-1·28) | 1·26 (1·14-1·38) | 1·35 (1·24-1·47) | 1·48 (1·37-1·60) |
| 6 | Reference | 1·12 (0·99-1·24) | 1·19 (1·07-1·32) | 1·28 (1·16-1·40) | 1·39 (1·27-1·52) |
| 6·5 | Reference | 1·07 (0·92-1·22) | 1·13 (0·98-1·28) | 1·22 (1·06-1·37) | 1·31 (1·16-1·47) |

# Table S6. Summary for the ratios of the modeled ADHD impairment scores for males with 95% confidence intervals.

|  | **Birth cohorts** | | | | |
| --- | --- | --- | --- | --- | --- |
| **ADHD symptom score** | **1994–1997** | **1998–2000** | **2001–2003** | **2004–2006** | **2007–2009** |
| 0 | Reference | NA | NA | NA | NA |
| 0·5 | Reference | 0·08 (-0·93-1·09) | 1·64 (0·62-2·66) | 2·79 (1·76-3·82) | 1·04 (0·01-2·07) |
| 1 | Reference | 0·53 (-0·01-1·07) | 1·41 (0·86-1·96) | 2·00 (1·44-2·57) | 1·07 (0·51-1·63) |
| 1·5 | Reference | 0·69 (0·31-1·06) | 1·35 (0·96-1·74) | 1·76 (1·35-2·17) | 1·10 (0·70-1·50) |
| 2 | Reference | 0·79 (0·52-1·06) | 1·32 (1·03-1·61) | 1·61 (1·30-1·92) | 1·13 (0·83-1·43) |
| 2·5 | Reference | 0·87 (0·67-1·07) | 1·30 (1·08-1·52) | 1·5 (1·26-1·74) | 1·15 (0·92-1·38) |
| 3 | Reference | 0·93 (0·78-1·09) | 1·28 (1·11-1·45) | 1·41 (1·22-1·61) | 1·17 (0·99-1·35) |
| 3·5 | Reference | 0·99 (0·86-1·12) | 1·27 (1·12-1·41) | 1·34 (1·18-1·51) | 1·19 (1·04-1·34) |
| 4 | Reference | 1·03 (0·90-1·15) | 1·25 (1·12-1·39) | 1·29 (1·14-1·44) | 1·20 (1·06-1·34) |
| 4·5 | Reference | 1·06 (0·94-1·19) | 1·24 (1·11-1·37) | 1·25 (1·11-1·38) | 1·22 (1·08-1·35) |
| 5 | Reference | 1·09 (0·96-1·23) | 1·23 (1·09-1·37) | 1·21 (1·07-1·35) | 1·23 (1·09-1·37) |
| 5·5 | Reference | 1·11 (0·97-1·26) | 1·22 (1·07-1·36) | 1·18 (1·05-1·32) | 1·24 (1·09-1·38) |
| 6 | Reference | 1·13 (0·98-1·28) | 1·21 (1·05-1·36) | 1·16 (1·02-1·31) | 1·24 (1·09-1·39) |
| 6·5 | Reference | 1·14 (0·99-1·30) | 1·20 (1·04-1·35) | 1·15 (1·01-1·29) | 1·25 (1·10-1·40) |
| 7 | Reference | 1·15 (1·00-1·31) | 1·19 (1·03-1·34) | 1·14 (1·00-1·29) | 1·25 (1·10-1·41) |
| 7·5 | Reference | 1·16 (1·00-1·32) | 1·18 (1·02-1·33) | 1·13 (0·99-1·28) | 1·26 (1·10-1·42) |
| 8 | Reference | 1·16 (1·01-1·32) | 1·17 (1·01-1·32) | 1·13 (0·99-1·28) | 1·26 (1·10-1·42) |
| 8·5 | Reference | 1·17 (1·01-1·32) | 1·16 (1·01-1·31) | 1·13 (0·99-1·27) | 1·26 (1·11-1·42) |
| 9 | Reference | 1·17 (1·02-1·32) | 1·15 (1·01-1·3) | 1·14 (1·00-1·28) | 1·27 (1·11-1·42) |
| 9·5 | Reference | 1·16 (1·02-1·31) | 1·15 (1·00-1·29) | 1·14 (1·01-1·28) | 1·27 (1·12-1·42) |
| 10 | Reference | 1·16 (1·02-1·31) | 1·14 (1·00-1·28) | 1·15 (1·01-1·29) | 1·27 (1·12-1·42) |
| 10·5 | Reference | 1·16 (1·01-1·31) | 1·13 (0·99-1·28) | 1·16 (1·02-1·30) | 1·27 (1·12-1·42) |
| 11 | Reference | 1·15 (0·99-1·31) | 1·13 (0·97-1·28) | 1·17 (1·01-1·32) | 1·27 (1·10-1·43) |
| 11·5 | Reference | 1·15 (0·96-1·33) | 1·12 (0·95-1·30) | 1·18 (1·00-1·36) | 1·27 (1·08-1·46) |
| 12 | Reference | 1·14 (0·93-1·35) | 1·12 (0·91-1·32) | 1·19 (0·98-1·40) | 1·27 (1·04-1·49) |
| 12·5 | Reference | 1·13 (0·88-1·39) | 1·11 (0·86-1·36) | 1·20 (0·94-1·46) | 1·27 (0·99-1·54) |

# Table S7. Summary for the ratios of the modeled ADHD inattention impairment scores for males with 95% confidence intervals.

|  | **Birth cohorts** | | | | |
| --- | --- | --- | --- | --- | --- |
| **ADHD symptom score** | **1994–1997** | **1998–2000** | **2001–2003** | **2004–2006** | **2007–2009** |
| 0 | Reference | NA | NA | NA | NA |
| 0·5 | Reference | 0·90 (0·62-1·17) | 1·22 (0·94-1·50) | 1·04 (0·75-1·32) | 0·96 (0·69-1·23) |
| 1 | Reference | 0·92 (0·71-1·12) | 1·19 (0·99-1·40) | 1·05 (0·84-1·27) | 0·96 (0·76-1·17) |
| 1·5 | Reference | 0·94 (0·80-1·09) | 1·17 (1·02-1·31) | 1·07 (0·91-1·22) | 0·97 (0·82-1·12) |
| 2 | Reference | 0·97 (0·86-1·08) | 1·14 (1·03-1·26) | 1·08 (0·96-1·20) | 0·99 (0·87-1·10) |
| 2·5 | Reference | 0·99 (0·89-1·10) | 1·12 (1·01-1·23) | 1·09 (0·98-1·20) | 1·01 (0·90-1·11) |
| 3 | Reference | 1·02 (0·91-1·13) | 1·11 (0·99-1·23) | 1·10 (0·99-1·22) | 1·03 (0·92-1·14) |
| 3·5 | Reference | 1·05 (0·93-1·17) | 1·10 (0·97-1·22) | 1·11 (0·99-1·23) | 1·06 (0·94-1·18) |
| 4 | Reference | 1·07 (0·95-1·19) | 1·09 (0·96-1·21) | 1·12 (1·00-1·24) | 1·09 (0·97-1·21) |
| 4·5 | Reference | 1·09 (0·98-1·21) | 1·08 (0·96-1·20) | 1·12 (1·01-1·23) | 1·12 (1·01-1·24) |
| 5 | Reference | 1·11 (1·01-1·21) | 1·08 (0·98-1·19) | 1·12 (1·02-1·22) | 1·15 (1·05-1·26) |
| 5·5 | Reference | 1·13 (1·03-1·22) | 1·08 (0·99-1·18) | 1·12 (1·03-1·22) | 1·19 (1·09-1·28) |
| 6 | Reference | 1·14 (1·03-1·25) | 1·09 (0·98-1·19) | 1·12 (1·02-1·23) | 1·22 (1·11-1·32) |
| 6·5 | Reference | 1·15 (1·00-1·30) | 1·09 (0·95-1·23) | 1·12 (0·98-1·26) | 1·24 (1·10-1·39) |

# Table S8. Summary for the ratios of the modeled ADHD hyperactivity/impulsivity impairment for males scores with 95% confidence intervals.

|  | **Birth cohorts** | | | | |
| --- | --- | --- | --- | --- | --- |
| **ADHD symptom score** | **1994–1997** | **1998–2000** | **2001–2003** | **2004–2006** | **2007–2009** |
| 0 | Reference | NA | NA | NA | NA |
| 0·5 | Reference | 0·90 (0·65-1·14) | 1·22 (0·97-1·47) | 1·04 (0·76-1·31) | 0·96 (0·69-1·23) |
| 1 | Reference | 0·92 (0·75-1·09) | 1·19 (1·01-1·37) | 1·05 (0·85-1·26) | 0·96 (0·76-1·16) |
| 1·5 | Reference | 0·94 (0·83-1·06) | 1·17 (1·04-1·29) | 1·07 (0·92-1·21) | 0·97 (0·83-1·12) |
| 2 | Reference | 0·97 (0·87-1·06) | 1·14 (1·04-1·24) | 1·08 (0·97-1·20) | 0·99 (0·87-1·10) |
| 2·5 | Reference | 0·99 (0·89-1·10) | 1·12 (1·02-1·23) | 1·09 (0·98-1·20) | 1·01 (0·90-1·12) |
| 3 | Reference | 1·02 (0·91-1·14) | 1·11 (0·99-1·23) | 1·10 (0·98-1·22) | 1·03 (0·91-1·15) |
| 3·5 | Reference | 1·05 (0·92-1·17) | 1·10 (0·97-1·22) | 1·11 (0·98-1·24) | 1·06 (0·94-1·18) |
| 4 | Reference | 1·07 (0·94-1·19) | 1·09 (0·96-1·22) | 1·12 (0·99-1·24) | 1·09 (0·97-1·22) |
| 4·5 | Reference | 1·09 (0·97-1·21) | 1·08 (0·96-1·21) | 1·12 (1·00-1·24) | 1·12 (1·00-1·24) |
| 5 | Reference | 1·11 (1·00-1·22) | 1·08 (0·97-1·20) | 1·12 (1·01-1·23) | 1·15 (1·05-1·26) |
| 5·5 | Reference | 1·13 (1·02-1·23) | 1·08 (0·98-1·19) | 1·12 (1·02-1·23) | 1·19 (1·08-1·29) |
| 6 | Reference | 1·14 (1·02-1·27) | 1·09 (0·97-1·20) | 1·12 (1·01-1·24) | 1·22 (1·10-1·33) |
| 6·5 | Reference | 1·15 (0·98-1·32) | 1·09 (0·94-1·25) | 1·12 (0·97-1·28) | 1·24 (1·08-1·41) |

# Table S9. Summary for the ratios of the modeled ADHD impairment scores for females with 95% confidence intervals.

|  | **Birth cohorts** | | | | |
| --- | --- | --- | --- | --- | --- |
| **ADHD symptom score** | **1994–1997** | **1998–2000** | **2001–2003** | **2004–2006** | **2007–2009** |
| 0 | Reference | NA | NA | NA | NA |
| 0·5 | Reference | -1·67 (-3·79-0·45) | -3·36 (-5·60--1·12) | -1·81 (-4·09-0·47) | -1·44 (-3·77-0·90) |
| 1 | Reference | 8·06 (1·79-14·33) | 14·82 (8·03-21·61) | 9·82 (2·96-16·68) | 8·46 (1·38-15·54) |
| 1·5 | Reference | 2·07 (0·96-3·18) | 3·72 (2·48-4·96) | 2·72 (1·48-3·96) | 2·41 (1·13-3·70) |
| 2 | Reference | 1·36 (0·85-1·87) | 2·40 (1·82-2·99) | 1·89 (1·31-2·47) | 1·70 (1·10-2·30) |
| 2·5 | Reference | 1·09 (0·80-1·38) | 1·88 (1·54-2·23) | 1·56 (1·22-1·09) | 1·42 (1·08-1·77) |
| 3 | Reference | 0·95 (0·76-1·14) | 1·61 (1·37-1·85) | 1·40 (1·16-1·63) | 1·28 (1·05-1·51) |
| 3·5 | Reference | 0·87 (0·71-1·03) | 1·44 (1·24-1·64) | 1·30 (1·11-1·48) | 1·20 (1·02-1·37) |
| 4 | Reference | 0·83 (0·68-0·97) | 1·33 (1·14-1·51) | 1·23 (1·06-1·40) | 1·14 (0·98-1·30) |
| 4·5 | Reference | 0·80 (0·65-0·95) | 1·25 (1·07-1·43) | 1·19 (1·02-1·36) | 1·11 (0·95-1·27) |
| 5 | Reference | 0·79 (0·64-0·94) | 1·19 (1·02-1·37) | 1·17 (0·99-1·34) | 1·09 (0·93-1·25) |
| 5·5 | Reference | 0·78 (0·63-0·94) | 1·16 (0·97-1·34) | 1·15 (0·97-1·33) | 1·08 (0·91-1·25) |
| 6 | Reference | 0·79 (0·63-0·94) | 1·13 (0·95-1·31) | 1·14 (0·96-1·32) | 1·07 (0·90-1·25) |
| 6·5 | Reference | 0·80 (0·64-0·95) | 1·11 (0·92-1·29) | 1·14 (0·96-1·33) | 1·07 (0·89-1·25) |
| 7 | Reference | 0·81 (0·66-0·97) | 1·10 (0·91-1·28) | 1·14 (0·96-1·33) | 1·07 (0·89-1·26) |
| 7·5 | Reference | 0·83 (0·68-0·98) | 1·09 (0·90-1·27) | 1·15 (0·96-1·34) | 1·08 (0·90-1·26) |
| 8 | Reference | 0·85 (0·70-1·01) | 1·08 (0·90-1·27) | 1·16 (0·97-1·35) | 1·09 (0·91-1·28) |
| 8·5 | Reference | 0·87 (0·72-1·03) | 1·08 (0·90-1·27) | 1·17 (0·98-1·36) | 1·10 (0·92-1·29) |
| 9 | Reference | 0·90 (0·74-1·06) | 1·09 (0·90-1·27) | 1·18 (0·99-1·38) | 1·12 (0·93-1·30) |
| 9·5 | Reference | 0·93 (0·76-1·10) | 1·09 (0·90-1·29) | 1·20 (1·00-1·40) | 1·13 (0·94-1·32) |
| 10 | Reference | 0·96 (0·78-1·14) | 1·10 (0·90-1·31) | 1·22 (1·01-1·43) | 1·15 (0·95-1·35) |
| 10·5 | Reference | 0·99 (0·79-1·19) | 1·11 (0·89-1·34) | 1·24 (1·00-1·47) | 1·17 (0·95-1·39) |
| 11 | Reference | 1·03 (0·79-1·26) | 1·13 (0·87-1·38) | 1·26 (0·99-1·53) | 1·19 (0·93-1·44) |
| 11·5 | Reference | 1·06 (0·78-1·34) | 1·14 (0·84-1·44) | 1·28 (0·96-1·60) | 1·21 (0·91-1·51) |
| 12 | Reference | 1·01 (0·76-1·44) | 1·16 (0·80-1·52) | 1·31 (0·92-1·69) | 1·23 (0·87-1·60) |
| 12·5 | Reference | 1·14 (0·72-1·56) | 1·18 (0·74-1·61) | 1·33 (0·86-1·80) | 1·26 (0·81-1·70) |

*Note.* Negative coefficients reflect the need for the third-degree polynomial to capture the curvature and inflections of the data, allowing the model to fit both upward and downward trends in the curve.

# Table S10. Summary for the ratios of the modeled ADHD inattention impairment scores for females with 95% confidence intervals.

|  | **Birth cohorts** | | | | |
| --- | --- | --- | --- | --- | --- |
| **ADHD symptom score** | **1994–1997** | **1998–2000** | **2001–2003** | **2004–2006** | **2007–2009** |
| 0 | Reference | NA | NA | NA | NA |
| 0·5 | Reference | 0·90 (0·63-1·170) | 1·22 (0·89-1·54) | 1·04 (0·76-1·31) | 0·96 (0·65-1·28) |
| 1 | Reference | 0·92 (0·72-1·11) | 1·19 (0·95-1·43) | 1·05 (0·85-1·25) | 0·96 (0·74-1·19) |
| 1·5 | Reference | 0·94 (0·81-1·08) | 1·17 (1·00-1·34) | 1·07 (0·92-1·21) | 0·97 (0·82-1·13) |
| 2 | Reference | 0·97 (0·86-1·08) | 1·14 (1·01-1·28) | 1·08 (0·96-1·20) | 0·99 (0·87-1·11) |
| 2·5 | Reference | 0·99 (0·88-1·11) | 1·12 (0·99-1·26) | 1·09 (0·96-1·22) | 1·01 (0·89-1·13) |
| 3 | Reference | 1·02 (0·90-1·15) | 1·11 (0·96-1·25) | 1·10 (0·96-1·24) | 1·03 (0·90-1·17) |
| 3·5 | Reference | 1·05 (0·91-1·18) | 1·10 (0·94-1·25) | 1·11 (0·96-1·26) | 1·06 (0·91-1·21) |
| 4 | Reference | 1·07 (0·93-1·21) | 1·09 (0·93-1·25) | 1·12 (0·96-1·27) | 1·09 (0·94-1·24) |
| 4·5 | Reference | 1·09 (0·95-1·23) | 1·08 (0·93-1·24) | 1·12 (0·97-1·27) | 1·12 (0·97-1·27) |
| 5 | Reference | 1·11 (0·98-1·24) | 1·08 (0·94-1·22) | 1·12 (0·98-1·26) | 1·15 (1·01-1·29) |
| 5·5 | Reference | 1·13 (0·99-1·26) | 1·08 (0·95-1·22) | 1·12 (0·99-1·25) | 1·19 (1·05-1·32) |
| 6 | Reference | 1·14 (0·99-1·29) | 1·09 (0·94-1·24) | 1·12 (0·98-1·26) | 1·22 (1·07-1·37) |
| 6·5 | Reference | 1·15 (0·95-1·35) | 1·09 (0·90-1·29) | 1·12 (0·94-1·31) | 1·24 (1·04-1·44) |

# Table S11. Summary for the ratios of the modeled ADHD hyperactivity/impulsivity impairment for females scores with 95% confidence intervals.

|  | **Birth cohorts** | | | | |
| --- | --- | --- | --- | --- | --- |
| **ADHD symptom score** | **1994–1997** | **1998–2000** | **2001–2003** | **2004–2006** | **2007–2009** |
| 0 | Reference | NA | NA | NA | NA |
| 0·5 | Reference | 0·90 (0·65-1·14) | 1·22 (0·98-1·46) | 1·04 (0·77-1·31) | 0·96 (0·72-1·20) |
| 1 | Reference | 0·92 (0·75-1·09) | 1·19 (1·03-1·35) | 1·05 (0·87-1·24) | 0·96 (0·80-1·13) |
| 1·5 | Reference | 0·94 (0·83-1·06) | 1·17 (1·05-1·28) | 1·07 (0·94-1·20) | 0·97 (0·86-1·08) |
| 2 | Reference | 0·97 (0·87-1·07) | 1·14 (1·04-1·24) | 1·08 (0·97-1·19) | 0·99 (0·89-1·08) |
| 2·5 | Reference | 0·99 (0·88-1·11) | 1·12 (1·01-1·24) | 1·09 (0·98-1·2) | 1·01 (0·90-1·12) |
| 3 | Reference | 1·02 (0·89-1·15) | 1·11 (0·98-1·24) | 1·10 (0·98-1·23) | 1·03 (0·90-1·16) |
| 3·5 | Reference | 1·05 (0·91-1·18) | 1·10 (0·96-1·24) | 1·11 (0·98-1·24) | 1·06 (0·92-1·20) |
| 4 | Reference | 1·07 (0·93-1·21) | 1·09 (0·95-1·23) | 1·12 (0·98-1·25) | 1·09 (0·95-1·23) |
| 4·5 | Reference | 1·09 (0·96-1·23) | 1·08 (0·95-1·22) | 1·12 (0·99-1·25) | 1·12 (0·98-1·26) |
| 5 | Reference | 1·11 (0·98-1·24) | 1·08 (0·95-1·21) | 1·12 (1·00-1·25) | 1·15 (1·02-1·29) |
| 5·5 | Reference | 1·13 (0·98-1·27) | 1·08 (0·94-1·23) | 1·12 (0·98-1·26) | 1·19 (1·04-1·33) |
| 6 | Reference | 1·14 (0·96-1·32) | 1·09 (0·90-1·27) | 1·12 (0·94-1·30) | 1·22 (1·04-1·39) |
| 6·5 | Reference | 1·15 (0·90-1·41) | 1·09 (0·84-1·35) | 1·12 (0·87-1·37) | 1·24 (0·99-1·50) |

# Table S12. Summary for the ratios of the modeled ADHD scores with 95% confidence intervals with ADHD symptoms scores truncated at 14.5.

|  | | | | | |
| --- | --- | --- | --- | --- | --- |
|  | **(1994,1997]** | **(1997,2000]** | **(2000,2003]** | **(2003,2006]** | **(2006,2009]** |
|  | | | | | |
| 0 | Reference | NA | NA | NA | NA |
| 0·5 | Reference | -0·41 (-1·77-0·95) | 3·13 (1·80-4·47) | 2·58 (1·25-3·92) | 0·34 (-1·00-1·69) |
| 1 | Reference | 0·37 (-0·25-0·99) | 2·06 (1·44-2·68) | 1·81 (1·18-2·43) | 0·79 (0·17-1·42) |
| 1·5 | Reference | 0·61 (0·22-0·99) | 1·74 (1·35-2·13) | 1·58 (1·18-1·98) | 0·95 (0·55-1·34) |
| 2 | Reference | 0·74 (0·48-0·99) | 1·57 (1·31-1·84) | 1·46 (1·18-1·74) | 1·03 (0·76-1·30) |
| 2·5 | Reference | 0·82 (0·65-1·00) | 1·46 (1·27-1·65) | 1·38 (1·18-1·58) | 1·09 (0·89-1·28) |
| 3 | Reference | 0·88 (0·76-1·01) | 1·38 (1·24-1·53) | 1·33 (1·18-1·48) | 1·13 (0·98-1·28) |
| 3·5 | Reference | 0·93 (0·83-1·03) | 1·33 (1·21-1·44) | 1·29 (1·17-1·42) | 1·16 (1·04-1·28) |
| 4 | Reference | 0·97 (0·88-1·05) | 1·28 (1·18-1·39) | 1·27 (1·16-1·37) | 1·18 (1·08-1·28) |
| 4·5 | Reference | 0·99 (0·91-1·08) | 1·25 (1·15-1·35) | 1·24 (1·15-1·34) | 1·20 (1·10-1·29) |
| 5 | Reference | 1·02 (0·93-1·11) | 1·22 (1·13-1·32) | 1·23 (1·13-1·32) | 1·21 (1·11-1·31) |
| 5·5 | Reference | 1·03 (0·94-1·13) | 1·20 (1·10-1·30) | 1·22 (1·12-1·31) | 1·22 (1·12-1·32) |
| 6 | Reference | 1·05 (0·95-1·15) | 1·18 (1·08-1·29) | 1·21 (1·10-1·31) | 1·23 (1·13-1·33) |
| 6·5 | Reference | 1·06 (0·96-1·17) | 1·17 (1·06-1·28) | 1·20 (1·09-1·30) | 1·23 (1·13-1·34) |
| 7 | Reference | 1·07 (0·96-1·18) | 1·16 (1·04-1·27) | 1·19 (1·08-1·30) | 1·24 (1·13-1·35) |
| 7·5 | Reference | 1·08 (0·97-1·20) | 1·15 (1·03-1·27) | 1·19 (1·08-1·30) | 1·24 (1·13-1·36) |
| 8 | Reference | 1·09 (0·97-1·21) | 1·14 (1·02-1·26) | 1·19 (1·07-1·30) | 1·25 (1·12-1·37) |
| 8·5 | Reference | 1·10 (0·98-1·22) | 1·14 (1·01-1·26) | 1·19 (1·07-1·31) | 1·25 (1·12-1·37) |
| 9 | Reference | 1·10 (0·98-1·22) | 1·13 (1·01-1·26) | 1·19 (1·06-1·31) | 1·25 (1·12-1·38) |
| 9·5 | Reference | 1·11 (0·98-1·23) | 1·13 (1·00-1·25) | 1·19 (1·06-1·31) | 1·25 (1·12-1·38) |
| 10 | Reference | 1·11 (0·99-1·23) | 1·13 (1·00-1·25) | 1·19 (1·06-1·31) | 1·25 (1·12-1·38) |
| 10·5 | Reference | 1·11 (0·99-1·24) | 1·12 (1·00-1·25) | 1·19 (1·07-1·32) | 1·25 (1·11-1·38) |
| 11 | Reference | 1·12 (0·99-1·24) | 1·12 (0·99-1·25) | 1·19 (1·07-1·32) | 1·24 (1·11-1·38) |
| 11·5 | Reference | 1·12 (0·99-1·25) | 1·12 (0·99-1·25) | 1·2 (1·06-1·33) | 1·24 (1·10-1·38) |
| 12 | Reference | 1·12 (0·98-1·26) | 1·12 (0·98-1·26) | 1·2 (1·06-1·35) | 1·24 (1·09-1·39) |
| 12·5 | Reference | 1·12 (0·97-1·27) | 1·12 (0·97-1·28) | 1·21 (1·05-1·36) | 1·24 (1·08-1·40) |
| 13 | Reference | 1·12 (0·95-1·29) | 1·13 (0·96-1·30) | 1·21 (1·04-1·39) | 1·23 (1·05-1·41) |
| 13·5 | Reference | 1·12 (0·92-1·32) | 1·13 (0·94-1·32) | 1·22 (1·01-1·42) | 1·23 (1·02-1·44) |
| 14 | Reference | 1·12 (0·89-1·35) | 1·13 (0·91-1·36) | 1·23 (0·98-1·47) | 1·23 (0·99-1·47) |
| 14·5 | Reference | 1·12 (0·85-1·39) | 1·14 (0·87-1·40) | 1·23 (0·95-1·52) | 1·22 (0·94-1·50) |
|  | | | | | |

*Note.* Negative coefficients reflect the need for the third-degree polynomial to capture the curvature and inflections of the data, allowing the model to fit both upward and downward trends in the curve.

# Figure S1. Comparisons of model fit between a cubic model and a local polynomial. For each birth cohort there is a mean ADHD impairment score with 95% confidence interval per ADHD symptom level.


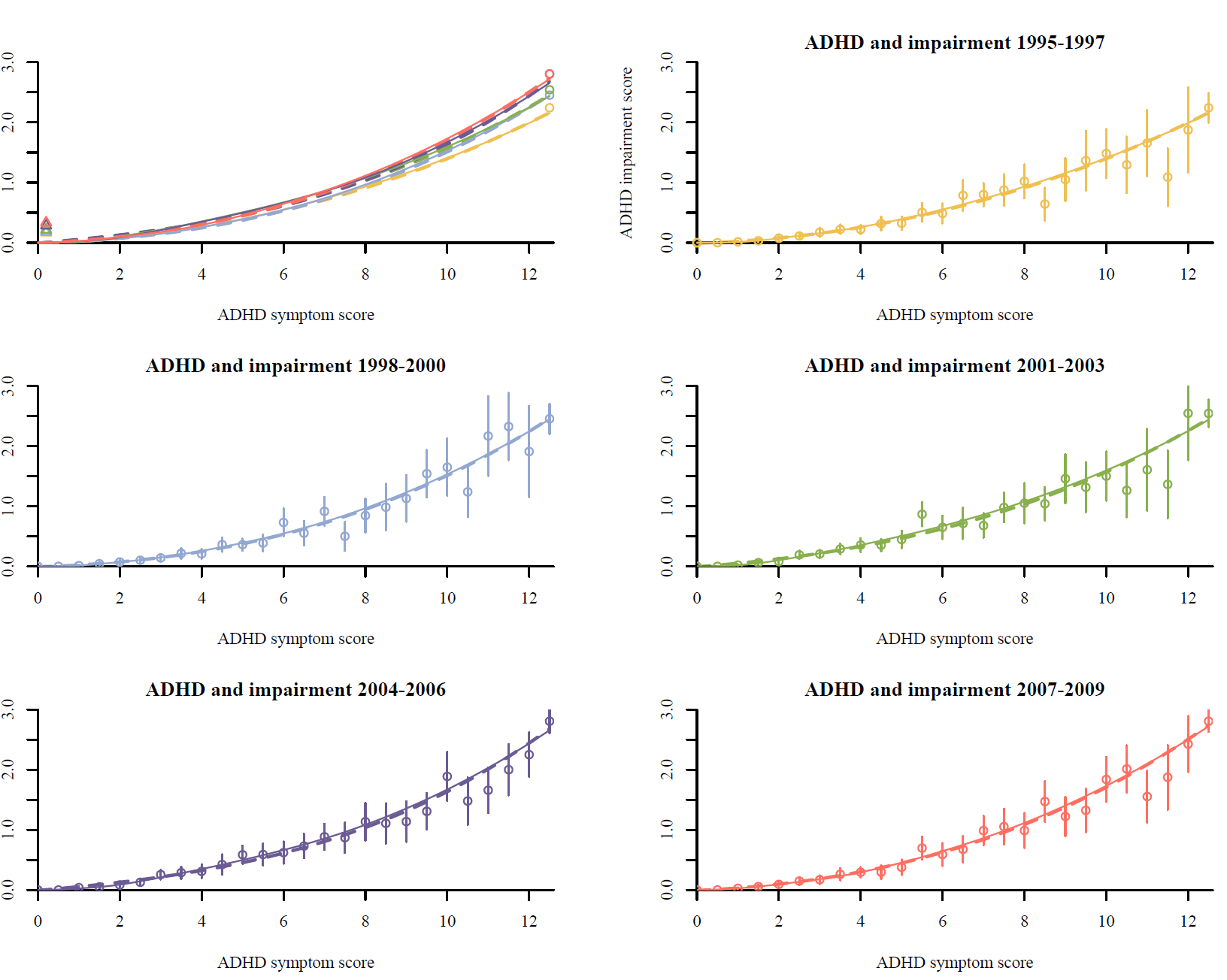


*Note.* Solid lines represent the cubic fit and dashed lines the local polynomial.

Appendix S1

|  | **Item No** | **Recommendation** | **Page No** |
| --- | --- | --- | --- |
| **Title and abstract** | 1 | (*a*) Indicate the study’s design with a commonly used term in the title or the abstract | 1 |
|  |  | (*b*) Provide in the abstract an informative and balanced summary of what was done and what was found | 2 |
| **Introduction** | | | |
| Background/rationale | 2 | Explain the scientific background and rationale for the investigation being reported | 4 |
| Objectives | 3 | State specific objectives, including any prespecified hypotheses | 4-5 |
| **Methods** | | | |
| Study design | 4 | Present key elements of study design early in the paper | 5 |
| Setting | 5 | Describe the setting, locations, and relevant dates, including periods of recruitment, exposure, follow-up, and data collection | 5-7 |
| Participants | 6 | (*a*) Give the eligibility criteria, and the sources and methods of selection of participants. Describe methods of follow-up | 5-7 |
|  |  | (*b*) For matched studies, give matching criteria and number of exposed and unexposed |  |
| Variables | 7 | Clearly define all outcomes, exposures, predictors, potential confounders, and effect modifiers. Give diagnostic criteria, if applicable | 6-7 |
| Data sources/ measurement | 8* | For each variable of interest, give sources of data and details of methods of assessment (measurement). Describe comparability of assessment methods if there is more than one group | 5 |
| Bias | 9 | Describe any efforts to address potential sources of bias | 5-8 |
| Study size | 10 | Explain how the study size was arrived at | 5-6 |
| Quantitative variables | 11 | Explain how quantitative variables were handled in the analyses. If applicable, describe which groupings were chosen and why | 5-8 |
| Statistical methods | 12 | (*a*) Describe all statistical methods, including those used to control for confounding | 7-9 |
|  |  | (*b*) Describe any methods used to examine subgroups and interactions |  |
|  |  | (*c*) Explain how missing data were addressed |  |
|  |  | (*d*) If applicable, explain how loss to follow-up was addressed |  |
|  |  | (*e*) Describe any sensitivity analyses |  |
| **Results** | | |  |
| Participants | 13* | (a) Report numbers of individuals at each stage of study—eg numbers potentially eligible, examined for eligibility, confirmed eligible, included in the study, completing follow-up, and analysed | 9 |
|  |  | (b) Give reasons for non-participation at each stage |  |
|  |  | (c) Consider use of a flow diagram |  |
| Descriptive data | 14* | (a) Give characteristics of study participants (eg demographic, clinical, social) and information on exposures and potential confounders | 9 |
|  |  | (b) Indicate number of participants with missing data for each variable of interest |  |
|  |  | (c) Summarise follow-up time (eg, average and total amount) |  |
| Outcome data | 15* | Report numbers of outcome events or summary measures over time | 9-10 |

| Main results | 16 | (*a*) Give unadjusted estimates and, if applicable, confounder-adjusted estimates and their precision (eg, 95% confidence interval). Make clear which confounders were adjusted for and why they were included | 9-10 |
| --- | --- | --- | --- |
|  |  | (*b*) Report category boundaries when continuous variables were categorized |  |
|  |  | (*c*) If relevant, consider translating estimates of relative risk into absolute risk for a meaningful time period |  |
| Other analyses | 17 | Report other analyses done—eg analyses of subgroups and interactions, and sensitivity analyses | 10 |
| **Discussion** | | | |
| Key results | 18 | Summarise key results with reference to study objectives | 10-11 |
| Limitations | 19 | Discuss limitations of the study, taking into account sources of potential bias or imprecision. Discuss both direction and magnitude of any potential bias | 11 |
| Interpretation | 20 | Give a cautious overall interpretation of results considering objectives, limitations, multiplicity of analyses, results from similar studies, and other relevant evidence | 11-12 |
| Generalisability | 21 | Discuss the generalisability (external validity) of the study results | 11-12 |
| **Other information** | | | |
| Funding | 22 | Give the source of funding and the role of the funders for the present study and, if applicable, for the original study on which the present article is based | 13 |

*Give information separately for exposed and unexposed groups.

**Note:** An Explanation and Elaboration article discusses each checklist item and gives methodological background and published examples of transparent reporting. The STROBE checklist is best used in conjunction with this article (freely available on the Web sites of PLoS Medicine at http://www.plosmedicine.org/, Annals of Internal Medicine at http://www.annals.org/, and Epidemiology at http://www.epidem.com/). Information on the STROBE Initiative is available at http://www.strobe-statement.org.
